# Detection of Memory Engrams in Mammalian Neuronal Circuits

**DOI:** 10.1523/ENEURO.0450-23.2024

**Published:** 2024-08-02

**Authors:** Nicole E. Niewinski, Deyanell Hernandez, Michael A. Colicos

**Affiliations:** Department of Physiology and Pharmacology, Cumming School of Medicine, Hotchkiss Brain Institute, University of Calgary, Calgary, Alberta T2N 4N1, Canada

**Keywords:** circuits, CNNs, engrams, memory, photoconductive stimulation, reverberatory loops

## Abstract

It has long been assumed that activity patterns persist in neuronal circuits after they are first experienced, as part of the process of information processing and storage by the brain. However, these “reverberations” of current activity have not been directly observed on a single-neuron level in a mammalian system. Here we demonstrate that specific induced activity patterns are retained in mature cultured hippocampal neuronal networks. Neurons within the network are induced to fire at a single frequency or in a more complex pattern containing two distinct frequencies. After the stimulation was stopped, the subsequent neuronal activity of hundreds of neurons in the network was monitored. In the case of single-frequency stimulation, it was observed that many of the neurons continue to fire at the same frequency that they were stimulated to fire at. Using a recurrent neural network trained to detect specific, more complex patterns, we found that the multiple-frequency stimulation patterns were also retained within the neuronal network. Moreover, it appears that the component frequencies of the more complex patterns are stored in different populations of neurons and neuron subtypes.

## Significance Statement

The existence of memory engrams, or reverberations of recently experienced activity patterns, has long been supposed but never directly demonstrated in mammalian neuronal networks. Through the use of highly accessible cultured neuronal networks grown on silicon wafers, stimulated to fire in user-defined patterns using photoconductive stimulation, we have demonstrated their existence and established a paradigm for the analysis of the microcircuitry involved.

## Introduction

The term “memory engram” was coined by Richard Semon in the early 1900s as a description of the imprint or storage of sensory stimulation in the brain (Semon, 1921). Scientifically pursued for over a century, the more system-level/psychological approaches consider the encoding, consolidation, and retrieval of a memory engram and include the classical works from Penfield and Milner (1958), Kandel (1976), Milner (1999), and a host of neuroscientists over the past century (for review, see Devan et al., 2018). The more mechanistic, empirical investigations arise from the classic works by Hebb (2005), Lashley (1950), Bliss and Lømo (1973), and again many others, where the mechanisms of synaptic plasticity, neuronal connectivity, and spatial distribution of memory have been studied. These studies, along with significant technical advances, have laid the foundation for breakthroughs in our study of the engram, such as engrams being mapped to cohorts of neurons (Rashid et al., 2016) and false memories implanted into animals (Ramirez et al., 2013). While current studies are revealing details on the architecture and behavior of the engram ensembles, these studies use correlative indicators of memory-related neuronal activity such as c-Fos expression (Jung et al., 2023, Ramsaran et al., 2023). In contrast, the goal of this study is to identify engram neurons by directly recording their activity after inputting user-defined firing patterns.

The concept of reverberating loops or circuits as a mechanism for short-term storage of information in the brain is almost as old as the engram and is best attributed to Hebb (2005). Often used in computational models of human memory (Niktarash, 2003), it is the concept that activity patterns from sensory experiences, for example, are held in repeating loops in neuronal circuits, while biochemical changes are occurring that will preserve the pattern for a longer period of time. Many of the core models of the mechanism of human memory embody a progression from a sensory experience to a pattern held in a reverberatory loop in a neuronal circuit, to a form of short-term memory (e.g., change in synaptic efficacy), and to long-term memory (e.g., permanent structural change).

The study of the underlying mechanisms and microcircuitry responsible for the information processing and storage performed by the brain extends across the breadth of neuroscience, and with recent technological advances, many in vivo paradigms are now accessible, and models of neuronal computation are being established (for example see Barron et al., 2020). However, the use of the more accessible networks in cultured neurons (Colicos et al., 2001; Potter and DeMarse, 2001) has proven viable models of learning and has even been suggested to possess biological intelligence (Kagan et al., 2022). In this study, our goal is to identify single-cell activity patterns reflecting the storage of experienced information, and to do so, we use high spatial and temporal monitoring of single-neuron response to computer-driven activity patterns.

## Materials and Methods

### Neuronal cultures

Experimental data were collected from primary dissociated cocultures of neurons and glial cells prepared from hippocampi of both female and male newborn (P0) Sprague Dawley rat pups (Charles River Laboratories), as previously described (Goda and Colicos, 2006). Briefly, neurons were plated on poly-D-lysine and laminin treated custom silicon wafers (1 cm^2^) at a density of 10^5^ cells/well in a 24-well plate. Previous experiments have demonstrated this to be an optimal density for providing robust activity while also mitigating burst firing from predominating the culture. Another factor helpful in reducing the chance of bursting in the network is to thoroughly triturate the cells before plating, to regulate the formation of neuronal aggregates which can play a role in modulating activity patterns (Okujeni et al., 2017; Montalà-Flaquer et al., 2022). In the referenced protocol, serum is reduced at a specific time after initial plating; however, this can also be optimized for your specific culture conditions. Reducing bursting activity aids in the detection of reverberations and in the training of neural networks from the activity patterns. Detection of engrams was optimal between 3 and 8 weeks of age and paralleled the maturation of the network. Thus, cultures were grown till ∼21 d in vitro (DIV) and subsequently used in experiments until ∼55–60 DIV. Experiments were performed on cultures from several different dissections (>8) to account for variability between animals and dissections. Cultures were maintained in a humidified, sterile incubator at 37°C with 5% CO_2_. Growth media consisted of Basal Medium Eagle supplemented with B27 and initially with 4% fetal bovine serum, which was decreased to 0% over 1 week. Subsequently, 50% of the growth medium was replaced twice a week with serum-free medium.

### Photoconductive stimulation and activity recording

Primary dissociated hippocampal cocultures were first incubated in a dark space for 20 min with 0.4 µM of the calcium indicator Fluo-4-AM and then transferred to a photoconductive stimulation device (chamber) containing a 37°C extracellular bath solution (EBS, osmolarity = 300–310 mOsm/L), pH = 7. Composition of the bath solution is as follows (in mM): 135 NaCl, 10 glucose, 3 CaCl_2_, 5 KCl, 1 MgCl_2_, 5 HEPES. The chamber was secured underneath the 10× objective lens of an upright Olympus BX51 microscope with a green fluorescent protein (GFP) light filter (488 nm) to observe the real-time calcium currents of illuminated Fluo-4-AM loaded neurons. The principle of photoconductive stimulation relies on the crystal structure of the silicon wafer on which the neurons are grown, which limits the increase in conductivity by light of the material to only the directly illuminated region. Both sides of the semiconductive silicon wafer are thinly insulated with silicon oxide, and by applying a charge to both sides (by way of electrodes in the bath solution), a capacitive charge is created on the wafer only in the illuminated region. This positive charge on the top surface of the wafer causes the negative ions in the cell to rapidly move to the bottom of the cell body, leaving a positive, depolarizing charge on the upper surface. This depolarization triggers an action potential to fire on the upper part of the membrane, and the subsequent opening of voltage-gated channels results in the entire cell being depolarized (Goda and Colicos, 2006). In practice, current is supplied by a Grass SD9 electrophysiology stimulator, controlled by a Tektronix Function Generator, which is in turn programmed through a serial interface with a PC running MATLAB to apply 2 ms firing pulses in the desired pattern or frequency. The chips were imaged for ∼5 min per region using a high-sensitivity CCD camera (Hamamatsu ORCA-Flash4) with a frame rate ranging from 30 to 100 fps depending on the experiment being performed. This ensures sufficient temporal resolution of neuronal activity and the stimulation pattern while minimizing acquisition time and subsequent processing time of datasets. Prior to recording, a small portion of the top-left corner of each chip was marked with clean forceps, and its coordinates were noted in reference to an electric motorized stage position. This allowed for proper positioning and region/cell identification in the network during subsequent imaging after immunostaining. The stimulation and activity recording paradigm begins with a 15–30 s low-frequency (0.2–0.5 Hz) electrical pulse to test the viability of individual neurons and the successful firing of these neurons by the device. This is referred to as the first test period which precedes a 60 s prestimulation period where spontaneous activity of the culture is recorded, acting as a control for stochastic firing of the culture. During the stimulation period, the neurons are fired in the desired pattern, and spontaneous activity is again recorded in the subsequent 60–90 s poststimulation period. Once the first experiment has been recorded from a chip, further experiments can be recorded on other distant regions of the same chip. After each movie, the position of the network is noted using the motorized stage, so it can be returned to for subsequent imaging.

### Determination of optimal parameters

**Figure 1.**
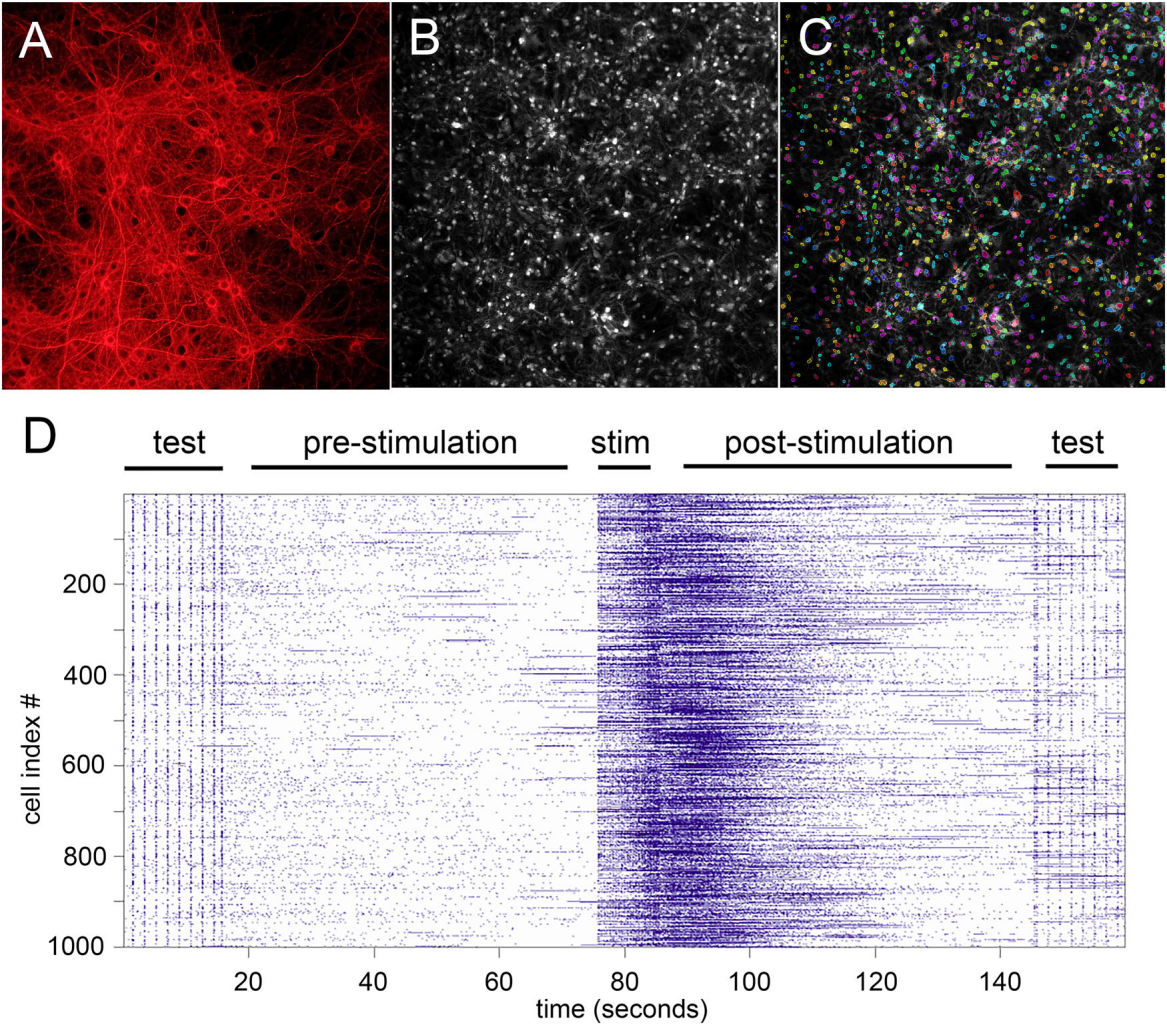
Engram recording stimulation paradigm. ***A***, MAP2 immunostaining of primary hippocampal cultures, illustrating the extensive network connections present. ***B***, Fluo4 calcium dye used to visualize neuronal firing. ***C***, Algorithm detection and indexing of cells. ***D***, Vertical axis gives the index number of the neuron traced, horizontal axis time. Following a test period (to determine functionally active neurons), a control prestimulation period of 60 s is recorded. Then a 10 s stimulation pattern is fired, followed by a poststimulation recording of 60 s. A final test period confirms the continued health of the neurons.

This project began as a single observation of an echo of a stimulation pattern being played back by a neuronal network after a stimulation event. As such, many parameters needed to be empirically determined to optimize this effect for rigorous study. The first consideration was to determine the best age for the culture. It is well known that the age, density, and growth conditions of primary neuronal cultures can radically alter their bursting behavior (Wagenaar et al., 2005, Bisio et al., 2014, Beer and Barak, 2024) and in terms of their criticality (Pasquale et al., 2008; Millman et al., 2010; Tyukin et al., 2019; Okujeni and Egert, 2023). A primary factor is the age of the culture. Relying on our experience working with primary neuronal cultures and corroborated by many studies in addition to the ones cited above, it has been observed that bursting activity in the neuronal network increases with age (and consequently connectivity) and is more likely to happen when the neurons are less evenly distributed across the growth substrate, perhaps due to the development of strong nodes of activity (personal observation). By optimizing the preparation methods and substrate (as described above), cultures that are less prone to bursting can be maintained to past 2 months of age. Such cultures have a stochastic appearance in the activity recording during the control period (for an example, see the prestim control period in Fig. 1). This makes observation of nonrandom patterns easier, and the greater age of the cultures also affords a higher degree of connectivity. Consequently, in our hands, the older (>1 month) cultures observably produced the reverberation effect more robustly. To determine the optimal single-frequency stimulation pattern, we tested the frequencies ranging from 3 to 10 Hz. Ten hertz was the maximum frequency found to be resolvable in the trace data using an exposure time of 20 ms, which was determined by a balance between the light level, exposure time, and health of the cells when illuminated. Results included demonstrated that the higher single frequencies were more effective in producing the reverberations. For the optimization of the multifrequency patterns, two variables were adjusted to increase the detection of reverberations of the stimulation pattern after the stimulation had ended. Two frequencies per pattern were present, a doublet with a specific interspike interval (ISI), and this doublet was repeated at a second, slower frequency. Timing between the two spikes of the doublet was initially varied in a range from 80 to 160 ms, which was based on historical studies of paired-pulse facilitation. Times for the repetition of the two-spike pattern ranged from 280 to 880 ms and were arbitrarily tested.

### Administration of inhibitors

The following drugs were used in the study and prepared as follows: APV stock 25 µM diluted in water, working conc. 100 µM; CNQX stock 10 µM diluted in DMSO, working conc. 100 µM; carbenoxolone stock 0.01 M diluted in water, working conc. 100 µM; suramin stock 0.01 M diluted in water, working conc. 100 µM; and KYNA stock 26.4 mM diluted in DMSO, working conc. 500 µM. Activity experiments were performed in the photoconductive stimulation device (described above) in EBS. After selecting a viable region of the network for the experiment, drugs were added directly to the ∼3 ml of EBS in the device. The solution is then gently agitated with a transfer pipet and allowed the appropriate time to incubate for the drug being tested.

### Controls

The most critical control for the induction of reverberations is the 60 s prestimulation recording period, where the baseline level of activity of the network is determined. During this time, the probability of the random occurrence of a stimulation pattern in the specific culture is quantified. To compare the random development over time of stimulation patterns, we performed the control experiments in which no stimulation was given, and these data are included as a separate column in the relevant graphs. Controls for the training of the recurrent neural networks (RNNs) were generated by multiple methods, including randomizing the ISIs of the actual training patterns, randomly generated noise, and recordings of spontaneous activity in control cultures. For statistical analysis, a large number of *n* were used as variance was in general high, and violin plots were used to convey the actual properties of the datasets. Data reported in figures show basic error bars for clarity; full statistical analysis of all data and additional control experiments can be found in Extended Data Figure 3*A*-1.

### Data processing

Once recordings have been made, data files were first converted to uncompressed AVI file format and processed through the public domain software NETCAL (http://www.itsnetcal.com). Analysis in this program entailed separating neuronal firing events from astrocytic calcium waves, spatial detection and indexing of individual neurons in the field of view (ROI), and extraction of their respective fluorescence traces. Various algorithms (OOPSI, OASIS, MLSPIKE, PEELING, FOOPSI) and respective parameters were evaluated and manually compared with calcium traces of numerous movies to identify which most closely represented spikes in calcium fluorescence. The FOOPSI algorithm was chosen as it was found to be the most accurate and consistent in inferring spikes from traces given a threshold that captures the maximum number of fluorescence spikes while avoiding detection of subthreshold noise. Glial calcium waves were excluded from the analysis at this point by virtue of their slow onset. This produced an array of discrete times indicating where each neuron had a spike in fluorescence.

### Data analysis

Manual programming in MATLAB was used for subsequent analysis on the output files from NETCAL. A binary raster plot was first produced to visualize inferred spikes of the neuronal population over time. Start and stop times for each region (test pattern, prestim, stim, and poststim periods) were manually identified at this stage. Thresholding methods were used to identify active cells in the network, which eliminated neurons that did not fire during a particular number of stim pulses. ISIs of firing cells were calculated from their inferred spikes through simple subtraction and grouped depending on whether they occurred before, during, or after the introduction of information via stimulation. To generate histograms, a bin size matching the exposure time of the recording was used for the generation of histograms (∼0.02 s + file processing time per frame, recorded during frame capture). The number of cells that were found to fire in the range of ±1 bin corresponding to the stimulation frequency was extracted individually for the prestimulation, stimulation, and poststimulation regions and compared. The percentage increase in the number of cells found to fire post- versus prestimulation was calculated by taking the number of cells firing in the poststim, subtracting that from the number found in the prestim and then that result was divided by the number of cells firing during stimulation. Comparing the prevalence of cells firing at the stimulation frequency before and after its introduction to the culture provides a method to quantify general changes in network activity relating to the input information.

### RNN analysis

Using the MATLAB (2021b) deep learning toolbox, an RNN was used to identify stimulation patterns in the poststimulation recordings. Briefly, the stimulation region from all active cells was used to generate a training set containing one-dimensional arrays that contained the firing pattern of one neuron for one cycle of the stimulation pattern. Therefore, in a typical experiment, there would be 10 times the number of active cells or ∼5,000 training samples for positive pattern identification. To generate the training set for negative pattern identification, we randomized the ISIs of the actual patterns. We also tested the random generated noise, as well as sequences taken from recordings during the prestimulation period and from recordings of random spontaneous activity. No significant difference in training effectiveness was noted between these methods. Once the training sets were created, the training of the network was initiated with the following parameters in general—optimizations were made depending on the nature of the specific experiment:

Layers = [
   sequenceInputLayer(1)
   bilstmLayer(64, “OutputMode”, “sequence”)
   bilstmLayer(64, “OutputMode”, “last”)
   fullyConnectedLayer(2)
   softmaxLayer
   classificationLayer
   ]
Options = trainingOptions(“adam”,…
   “MaxEpochs”, 10,000,…
   “MiniBatchSize”, 1,000,…
   “InitialLearnRate”, 0.005,…
   “SequenceLength”, 100,…
   “GradientThreshold”, 0.99,…
   “ExecutionEnvironment”, “auto”,…
   “plots”, “training-progress”,…
   “Verbose”,true);

The training was considered successful when the error rate was close to zero, and prediction testing was 100% accurate with the training patterns. To analyze a full activity fingerprint, activity signals from each neuron were run through the RNN in a sliding window to predict if each point in the trace was either a match to the stimulation pattern or not and the degrees of confidence used to create the output seen in Figure 4.

### Immunocytochemistry

Directly after the activity recording paradigm, the silicon chip was fixed at room temperature for 30 min in a 4% paraformaldehyde solution prepared as previously described (Goda and Colicos, 2006). This was followed by a 5 min wash in 1× phosphate-buffered saline (PBS) and then refrigerated in PBS until immunostaining was performed. For subsequent immunocytochemical analysis, the fixed chip is washed three times in 1× PBS for 5 min each and left in a 2% bovine serum albumin blocking solution in PBS containing 5% goat serum, 5% donkey serum, and 0.25% Triton at room temperature for 1 h. The chip was washed; this and all subsequent washing periods involved three 5 min washes in 1× PBS. A refrigerated application of the primary antibody solution then occurred overnight, with the solution consisting of anti-VGLUT2 (1:200, Synaptic Systems, 135404) and anti-GABA (1:500, Sigma-Aldrich, A2052) diluted in blocking solution. Afterward, the chip was washed and placed into a secondary antibody solution consisting of Alexa Fluor 647 (1:1,000) and Alexa Fluor 488 (1:1,000) diluted in a blocking solution for 1 h at room temperature in a dark space to prevent photobleaching. The chip was washed again and then placed directly into the photoconductive stimulation chamber with EBS such that it was oriented corrected with the corner marked during experiments in the top left. The chamber was placed underneath the microscope, and coordinates of the top-left corner were taken again. Thus, any discrepancies between the initial and current corner coordinates due to differing placement of the chamber underneath the microscope were accounted for. The coordinates of each of the movies previously filmed on that chip were calculated. Once a region has been located, GFP and CY-5 light filters were used to visualize VGLUT2-positive and GABA-positive neurons, respectively. Light levels were adjusted to minimize background noise and maximize the fluorescence of stained cells. Multiple images were taken with an exposure time of 1 s over a period of 4 s to create a single high-resolution composite image using each of the two filters. The two images were then overlayed with green or red filters in the program ImageJ to represent VGLUT-positive and GABA-positive neurons, respectively. Then the two-colored images were overlayed themselves to create a single composite image. To match the trace and spike data from each of the neurons to a cell type, the ROI locations are overlayed on top of the composite image. From here, each cell is manually labeled as being excitatory for VGLUT2-positive neurons or inhibitory for neurons positively stained for GABA. This allows for spatial mapping of the engram cells and for statistical analysis to determine the contribution of cell identity in the storage of information within the neuronal circuit. The Excel file is then imported into MATLAB to investigate potential correlations between cell identity and other factors related to the cells and circuitry that regenerated the stimulation frequency.

## Results

Figure 1 illustrates the experimental paradigm. Hippocampal neurons from newborn rat pups are cocultured with glial cells on silicon wafers, where they rapidly grow and connect to form complex networks (Fig. 1*A*). The cultures are environmentally supported and grown for 4–8 weeks and then stained with the calcium indicator Fluo-4-AM (Fig. 1*B*), allowing active cells to be easily identified and indexed [Fig. 1*C*; see Colicos et al. (2001) and Materials and Methods for details]. Once the cultures have matured to the desired time point (usually 4–6 weeks is optimal), the silicon wafers with the neurons are placed acutely in the photoconductive stimulation apparatus. This device functions to noninvasively stimulate only the neurons that are illuminated, the mechanism by which (capacitive depolarization) has been well documented (Colicos et al., 2001; Goda and Colicos, 2006; Hung and Colicos, 2008; Gutierrez et al., 2009; Pavlov et al., 2010). Figure 1*D* illustrates a typical stimulation paradigm. Neuronal networks are allowed to stabilize in the bath solution, and then several widely spaced pulses (0.5 Hz) are delivered to the cells, stimulating them to fire. This allows confirmation of the health and ability of the neurons to fire in response to stimulation. Then a 1 min control period is recorded during which time no stimulation is applied, followed by 10 s of stimulation at the desired frequency or pattern and then after the stimulation is turned off 60 s of recording to capture the response of the network. After the analysis period is over, the slow test pattern is repeated, to confirm the health of the neurons being tracked. This is referred to as the standard test conditions. It can be seen in Figure 1*D* that stimulation results in an increase in activity that persists following the cessation of stimulation and that this increase is transitory. It is during this poststimulation activity that we investigated the presence of reverberations of the stim pattern.

**Figure 2.**
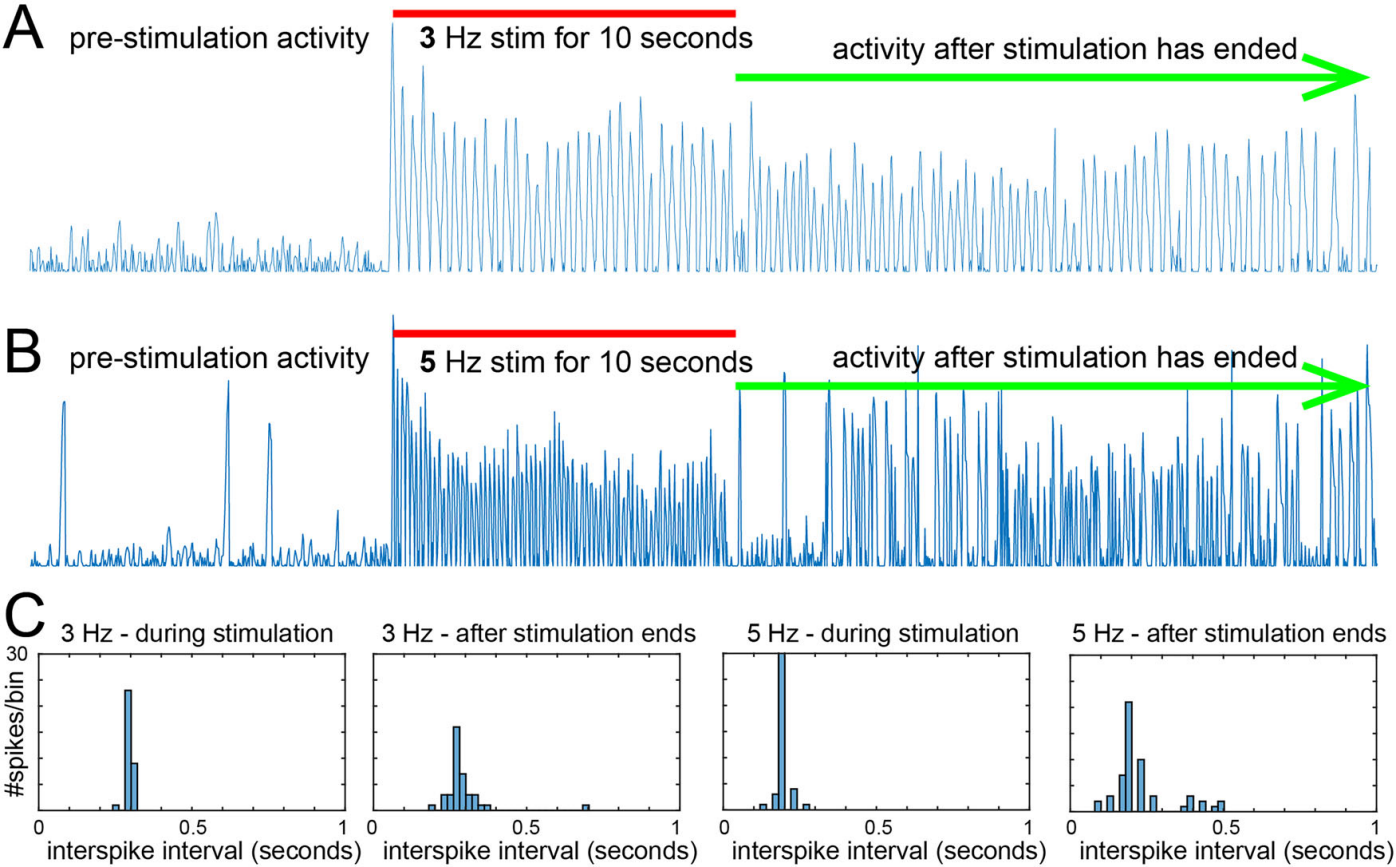
Identification of reverberations of the stimulation frequency after the stimulation has ended. ***A***, The trace shows the calcium signal recording from a single neuron in the network. During the prestim period, background noise and minor depolarizations can be seen, and during the 3 Hz stimulation (red bar), the neuron fires reliably. After the stimulation has ended (green arrow), firing continues at a frequency resembling the stimulation frequency. ***B***, The trace shows the same experiment with a 5 Hz stim frequency instead. ***C***, Sample histograms of ISIs show a clear peak at the wavelength of the stim frequency (either 3 or 5 Hz) during the stimulation, as expected. This peak is also evident, along with other frequencies, during the poststimulation period. Quantification of the prevalence of these reverberations will be presented in Figure 3. The baseline of calcium traces normalized for visualization.

To detect reverberations of specific neuronal activity patterns that persist following a stimulation event, we first used simple single-frequency stimulation patterns. Mature cultures, between 32 and 48 DIV were run through the standard test conditions (see above) using either a 3 or a 5 Hz stimulation for 10 s. It can be seen from Figure 2 that while only random stochastic spontaneous neuronal firing occurred prior to stimulation, after the stimulation stopped, some neurons continued to autonomously fire at the stimulation frequency. This could be directly detected with the lower 3 Hz stimulation (Fig. 2*A*) but also appeared to be present with the higher 5 Hz stimulation (Fig. 2*B*). To quantify the presence of stimulation pattern-specific reverberations, histograms of the ISI during the poststimulation were generated. The first panel in Figure 2*C* shows the ISI calculated from the population of neurons which had confirmed viability by their ability to fire during the test period. As can be seen in the example neuron in Figure 2*B*, the firing events during stimulation are very regular as expected and produce a histogram strongly centered on a 0.33 s peak. The second panel shows the ISI distribution during the poststimulation period; again with a peak at this duration, it is broader and less pronounced but clearly present. The third and fourth panels show the same analysis for the 5 Hz stimulation, with similar results. These results suggest the presence of reverberations of the single-frequency stimulation, which was then substantiated by determining the frequency of occurrence of this phenomenon.

**Figure 3.**
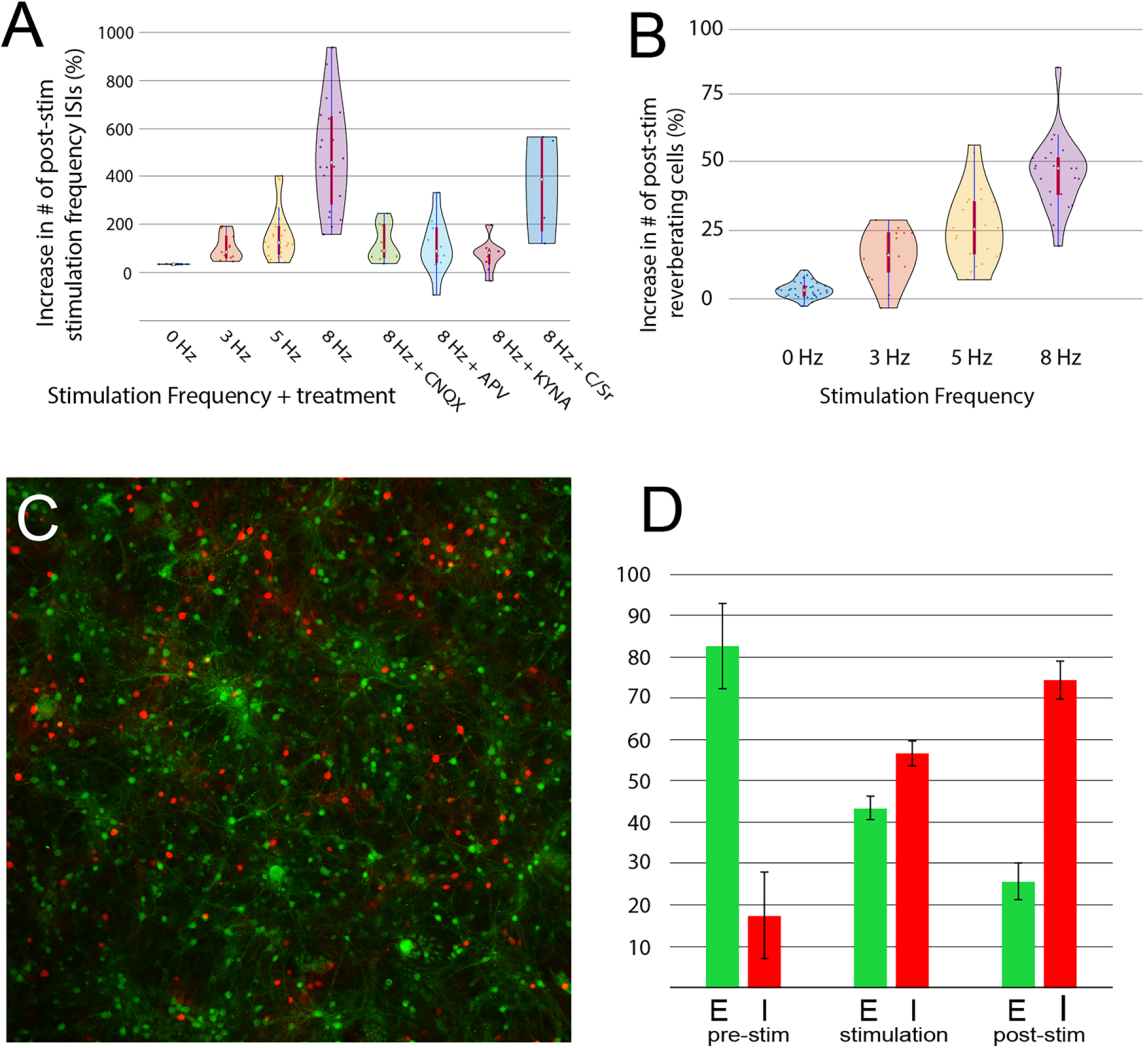
Frequency of occurrence of reverberations, dependency on synaptic transmission, and neuronal cell types involved. ***A***, The percentage increase in ISIs corresponding to the stimulation frequency in the 60 s poststimulation versus the prestimulation control period. Zero hertz represents a nonstimulated control. A 3, 5, and 8 Hz stimulation results in an increasing presence of stimulation-specific reverberations. Glutamatergic synaptic transmission blockers CNQX, APV, and KYNA reduce the reverberations at 8 Hz, while the glial transmission blockers carbenoxolone and suramin also demonstrate that glia could play a role in the process. ***B***, Data in ***A*** show the total amount of reverberations seen in the network, while ***B*** indicates the increase in the percentage of the population of individual cells that have reverberations. Again, an increase with frequency is observed. ***C***, Costaining of excitatory (VGLUT) and inhibitory (GABA) neurons, which is then cross-referenced to each cell's activity (Fig. 1*C*,*D*). ***D***, Neuronal type of cells expressing reverberations. In the prestim control period, a predominance of excitatory activity is observed, as expected in a normal active network. During the stimulation, all cell types are fired equally, and so the *E*/*I* ratio reflects the composition of the network. Poststimulation however, a strong skew to inhibitory cell activity is observed, suggesting inhibitory cells are an important component of the microcircuitry responsible for the reverberations. Full statistical analysis and discussion regarding controls in Extended Data Figure 3*A*-1, Table 3*C*-1*a,b*,*D*-1.

Data analysis was performed to determine both what percentage of the neurons in the network exhibits this behavior and how often the reverberations occur in the population as a whole. We also determined what cell types expressed this behavior, in terms of excitatory or inhibitory neurons, and the dependency on synaptic transmission (Fig. 3). Experiments were performed as described in Figure 1. As a control, the network was stimulated at “0 Hz” (meaning no stimulation; for a further description of control analysis, see Extended Data Fig. 3*A*-1), and the number of ISIs at the frequency most effective in producing the reverberations (8 Hz; see below) was compared with their presence before and after the 10 s midpoint of the recording (Fig. 3*A*, first column). As expected, there was no significant change in the occurrence of individual frequencies (*n* = 5). To control for the random occurrence of neurons firing at the same frequency as the stimulation, we determined the number of ISIs of the corresponding wavelength during a 60 s control period before the stimulation, and this was then used to calculate the percentage increase in the occurrence of reverberations of this frequency poststimulation. This value was also normalized for the number of neurons actually firing in the network, as determined by a positive response to the test pulses at the start of the experiment (Fig. 1). Figure 3*A* shows that while a 3 Hz stimulation resulted in a 55% increase (±13% SEM, *n* = 16), a 5 Hz stimulation pattern produced a 123% increase (±23% SEM, *n* = 23), and an 8 Hz stimulation increased this to an over 500% increase (513 ± 90% SEM, *n* = 22).

10.1523/ENEURO.0450-23.2024.f3A1 Figure 3 **Language genetic influence result without a language mask** **A. Multiple types of whole-brain regions.** After genetic modeling without restricting the language activation map, multiple types of regions were identified. **B. The clustering result of the genetic regions without a language activation mask**. Download Figure 3, TIF file.

10.1523/ENEURO.0450-23.2024.t3A1a Table 3A- 1a **Functional labels based on Neurosynth for genes contributing to commonality of language clusters** Note: For each gene, the functional terms from Neurosynth represent the terms with the most similar meta-analysis whole-brain activation map to the gene’s whole-brain map. The correlation values indicate the correlation of the gene’s whole-brain expression with the term’s meta-analysis result. Download Table 3A, DOC file.

10.1523/ENEURO.0450-23.2024.t3A1b Table 3A - 1b **Functional labels based on Neurosynth for genes contributing to differences of different language clusters** Note: For each gene, the functional terms from Neurosynth represent the terms with the most similar meta-analysis whole-brain activation map to the gene’s whole-brain map. The correlation values indicate the correlation of the gene’s whole-brain expression with the term’s meta-analysis result. Download Table 3A - 1b, DOC file.

10.1523/ENEURO.0450-23.2024.t3D1 Table 3D - 1 Download Table 3D - 1, DOC file.

It is uncommon for mammalian hippocampal neurons to fire tonically at specific frequencies under physiologically unperturbed conditions without being part of a synaptic circuit (or through autaptic connections; Siebler et al., 1993). However, to demonstrate that the reverberations are part of a microcircuit between more than one neuron, we repeated the experiments at 8 Hz in the presence of synaptic transmission inhibitors. It can be seen in Figure 3*A* that blocking either AMPA receptors (with 100 µM CNQX) or NMDA receptors (with 100 µM APV) both could greatly reduce the production of the reverberations (513% increase reduced to 60 ± 27% SEM, *n* = 8, for CNQX and to 68 ± 34% SEM, *n* = 8, for APV) and that KYNA (500 µM, which blocks all glutamatergic transmission) reduces the effect even more (to 37 ± 21% SEM, *n* = 9). This dramatic reduction indicates that the stored reverberations are dependent on synaptic transmission and suggest that they are a product of a circuit or microcircuit in the network. As current research has supported a greater role of glial cells in the modulation of synaptic transmission (for review, see Liu et al., 2023), we also tried blocking gap junctions (with carbenoxolone, 100 µM) and P2 receptors (with suramin, 100 µM). While the results had a high degree of variance (332 ± 113% SEM, *n* = 4), they do suggest that normal signal transmission between glial cells could be important for the reverberations to occur.

Figure 3*A* reports the prevalence of the reverberations of the stimulation frequency in the network as a whole; however, the number of neurons in the network which participate in the phenomenon is also of interest. We observed that a 3 Hz stimulation results in a ∼14% (±2% SEM, *n* = 16) increase in the number of cells firing at that frequency and that 5 and 8 Hz stimulation produced ∼25% (±3% SEM, *n* = 24) and ∼46% (±3% SEM, *n* = 24) more cells firing at those specific rates (Fig. 3*B*). To determine what neuronal cell types are reverberating at the stimulation frequency, following the recordings we indexed the regions of the cultures recorded and then fixed the networks and used immunohistochemistry for inhibitory (GABAergic, Sigma-Aldrich, A2052) and excitatory (VGLUT2+, Synaptic Systems, 135404) neurons to identify the cell type (Fig. 3*C*). After returning to the region of the recording, the wafers were imaged and referenced back to the recorded ROIs (Fig. 1*C*,*D*). Interestingly we found that ∼75% of the neurons recruited to fire at the stimulation frequency were inhibitory. Figure 3*D* shows the dynamics of this recruitment. During the prestimulation control period, the network activity consists predominantly of firing excitatory neurons (82.5 ± 10.3% SEM, *n* = 3 excitatory); then during the stimulation (when all neuronal cell types are stimulated equally), there is a relatively equal number of excitatory and inhibitory neurons firing (43.4 ± 5.1% SEM, *n* = 3 excitatory), which reflects the neuronal composition of the culture. However, after the stimulation has ended, the majority of the neurons firing at the stimulation frequency (not in the network as a whole) are inhibitory (25.7 ± 7.8% SEM, *n* = 3 excitatory). This suggests that the reverberations of the stimulation pattern are predominantly stored in inhibitory neurons. Full statistical analysis of all data in Figure 3 can be found in Extended Data Table 3*A*-1*a,b*,*D*-1.

Taken collectively, these data demonstrate that a stochastically firing neuronal network stimulated at a specific frequency for a short time will continue to fire at that frequency after the stimulation is stopped. The presence of these reverberations of the experienced firing pattern in the network as a whole is more abundant at higher frequencies and is accompanied by the recruitment of new neurons rather than just an enhancement of activity in already firing cells. While the overall number of active neurons increases, the majority of the neurons specifically participating in the reverberations are inhibitory.

**Figure 4.**
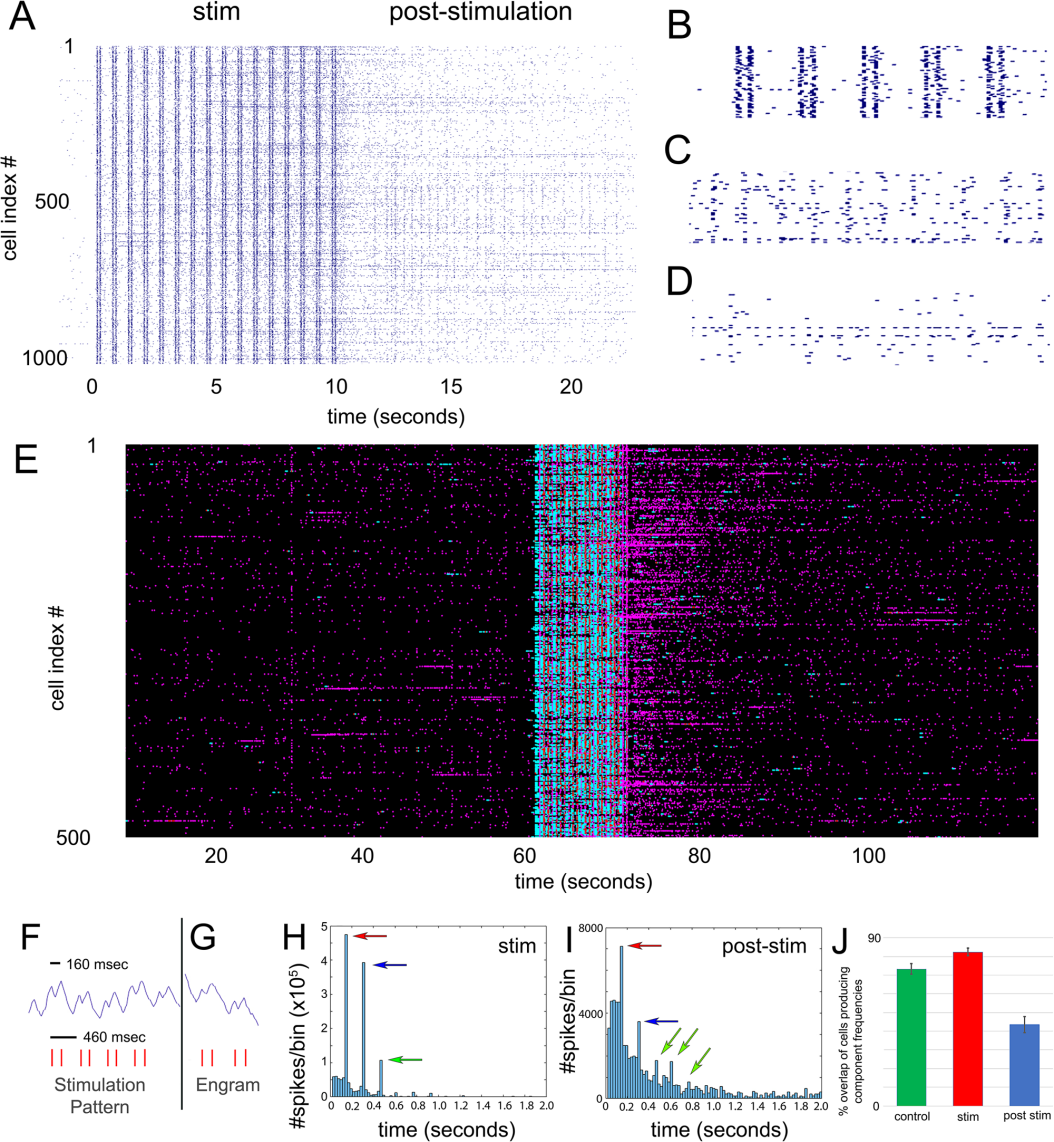
Engrams of multifrequency stimulation patterns; RNN, ISI, and circuitry analysis. Patterns containing two component frequencies are used to briefly stimulate the network. ***A***, Overview of the 10 s stim and the network activity immediately afterward in ∼1,000 neurons. Faint vertical bands can be seen to persist after stimulation has ended. ***B***, Close-up of the stim pattern. ***C***, ***D***, Close-up of two regions in the poststim region, suggesting some cells have low-frequency activity (***C***), and some have higher-frequency firing patterns (***D***). ***E***, RNN identification of the stimulation pattern in the entire activity fingerprint. Brightness indicates the degree of similarity to the stim pattern, which was used to train the network. The pattern itself (at 60 s) is the strongest region identified as expected; however, many regions of higher similarity can be seen immediately following the stim in comparison with the prestimulation region. RNN statistics in text. While the RNN analysis produced superior identification of complete engram patterns, histogram analysis allows a more direct analysis of the component frequencies. ***F***, AN example of a single-neuron firing trace during stimulation, illustrating the two component frequencies. ***G***, An example of a neuron in the poststim region repeating a similar pattern. ***H***, Histogram analysis of the ISIs during the stimulation of the entire network. Similar to what we saw with the single frequency, peaks at frequencies corresponding to the high-frequency component (red, 160 ms) and the ISI between the end of one doublet and the start of the next (blue, 300 ms) can be seen, as well as a population at 460 ms (green), the distance from the start of one full pattern to the next. ***I***, Histogram analysis of the poststim region. While the 160 and 300 ms components are detectable, other peaks are frequently observed (green), including the interpattern interval of 460 ms. ***J***, Comparison of the neuron populations in the high-frequency (160 ms) versus low-frequency (460 ms) components. Under nonstimulated, spontaneous network activity, we found a 73.3 ± 2.85% SEM (*n* = 8) overlap between the neurons, meaning many cells were firing at both frequencies. During stimulation, this number was higher (82.3 ± 2.0%, SEM *n* = 12), which was expected as all neurons in the network are being driven to fire at both frequencies. In the poststimulation period, there was a substantial decrease in the overlap (43.6 ± 4.3%, *n* = 15), suggesting different populations of neurons were firing at the two different component frequencies. Full statistical analysis in Extended Data Table 4*J*-1.

After establishing the persistence of reverberations of a simple single-frequency stimulation, we then increased the complexity of the experienced pattern by including two distinct frequencies during the stimulation. Figure 4*A* illustrates a short time window of the neuronal activity during such a stimulation protocol, showing the 10 s stimulation pattern and ∼12 s afterward. In this example, the vertical bands of synchronous firing that continue to occur after the stimulation ends are noteworthy, which bear some resemblance to the stim pattern itself. Figure 4*B* shows a close-up of a region of the stimulation pattern, which is delivered with the fast frequency of 100 ms, staggered by 400 ms between the start of each doublet. At this level of detail, it can be seen that other spontaneous firing activity continues to occur, which is by design as the stimulation is meant to overlay the normal activity in the network. Figure 4, *C* and *D*, shows two close-ups from the poststimulation region, where in Figure 4*C* it appears that the lower frequency is being repeated by neurons and in Figure 4*D* the higher frequency is being repeated. To quantify these events, while histogram analysis is fine for the single component frequencies, accurately detecting the complete pattern required a different approach. Figure 4*E* illustrates the methodology used (details in Materials and Methods). As it is well suited for sequence and time-series classification, a RNN was trained with the stimulation pattern as the target (Category 1) and a randomized version of the same ISIs as a second category (Category 0). Training of the RNN was performed until a high (>99%) level of accuracy was achieved with the target stim sequence as a test. We then used the RNN to test the entire activity fingerprint (e.g., a dataset as illustrated in Fig. 1*D*), going through each cell's firing sequence individually in small steps (10–50 ms time points each shift). The similarity of each sample was plotted back on the original graph scale, an example of which is seen in Figure 4*E*. As an indication of its effectiveness, it can be seen that the stimulation region (between 60 and 70 s) is detected very strongly. Some regions in this example in the prestimulation control region can also be seen as positive; however, far more positive regions can be identified in the poststimulation region. Statistically, we found a 737% increase (±81%, *n* = 6) in the number of engrams poststimulation. As we are identifying a complex pattern in its entirety, we refer to these as engrams rather than reverberations. These data suggest that complex stimulation patterns are also maintained after they are experienced by the network.

10.1523/ENEURO.0450-23.2024.t4J1 Table 4J - 1 **Genetic model results for all the language genetic clusters after excluding specific object domain responsive voxels** Note: For each language genetic cluster, the cognitive abilities with existing genetic effects (AE model or DE model, p values < 0.05, compared with the control model E, uncorrected) are marked with yellow. ΔAIC denotes the degree to which the best model is better than the control model. Download Table 4J - 1, DOC file.

To investigate the specific neuronal populations that are expressing the complex patterns, we once again employed a basic histogram analysis of the ISI between neuronal firing events in each neuron. Illustrated in Figure 4*F*, an example of one such pattern can be seen on the left panel—pairs of pulses 160 ms apart, repeated at 460 ms intervals again for 10 s total. While we could visually identify neurons that expressed the complete pattern after the simulation was stopped (Fig. 4*G*), quantification of these more complex patterns overlaid with other neuronal activity was challenging, and so we first identified the component frequencies as was performed in the single-frequency experiments. Figure 4*H* shows the histogram distribution seen during the stimulation, with three well-defined peaks observed; the first peak corresponds to the highest frequency component of 160 ms (red arrow; Fig. 4*F*); the second peak corresponds to the ISI between the start and end of the high-frequency doublet (blue arrow, 460–160 = 300 ms); and the third smaller peak corresponds to the distance between the repeats of the pattern (green arrow, 460 ms). In the case of the stimulation, where all neurons are being driven to fire by the stimulation device, this ISI is presumably caused by neurons that failed to fire either the second of the high-frequency doublets in one pattern repeat or the first of the doublets of the next repeat. Looking in the poststimulation region (Fig. 4*I*), we observe the presence of primarily the 160 ms ISI component (corroborating the RNN results), but we also see evidence of 300 ms, 460 ms, and other lower-frequency peaks as well (green arrows). While the general increase in network activity renders this analysis method inferior to the RNN for distinguishing engrams, it does allow the identification of the specific neurons that produce the component frequency of a multifrequency stimulation pattern. Using these data, we determined which neurons were producing the high- and which were producing the low-frequency components and calculated the degree of overlap between the two populations. Figure 4*J* shows the results of this; first, a control was taken from multiple recordings of spontaneous network activity made before any experimentation. Under nonmanipulated conditions, we see a ∼70% overlap between the neuronal population producing the two different frequencies. During the stimulation, this increases to ∼80%, which is anticipated because the network is being exogenously stimulated to fire all neurons at these frequencies. Remarkably, however, the overlap between the populations drops to below 50% when the engrams are being produced in the poststimulation period. This observation supports the hypothesis that the subcomponents of the more complex dual-frequency pattern is stored in different populations of cells and reflects the qualitative observations made of these different populations in Figure 4, *C* and *D*.

## Discussion

These experiments demonstrate several important inherent qualities of mammalian neuronal networks. First, we show that reverberations of specific simple activity patterns received by a network can persist after the stimulation has ended. The ability to “hold” a pattern in the brain's neuronal circuits would provide a short-term or working memory mechanism. In our case, the network is “remembering” a specific frequency it experienced. This in itself is an important observation, in that it has long been assumed to exist, yet there has been a lack of direct evidence that it does. Our data also suggest this process has an underlying microcircuitry in which different neuronal cell types are differentially implicated in the process, in that inhibitory activity increases from a level that was previously less than that of excitatory cells, a finding in itself which suggests several simple circuits made from a pair of neurons which could function like this come into play (e.g., see schematic, below).

**Figure:**
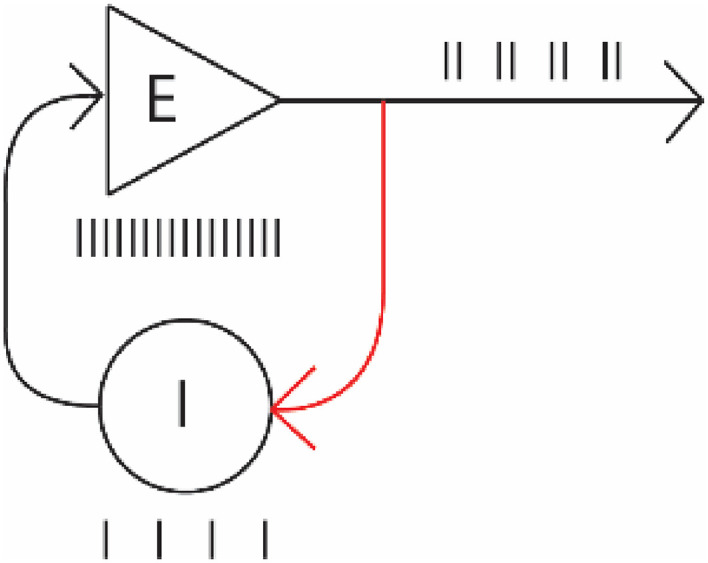


Second, we show that more complex patterns (with two component frequencies) can be retained in the network as well. As these are more difficult to identify, we developed a recursive neural network, trained on the stimulation pattern itself, which successfully identified the pattern during the stimulation event itself but also could be used to quantify the presence of the engram of that pattern in the poststimulation period. Although not as effective in the detection of complex patterns, examination of the poststimulation network activity by simple histogram analysis of the ISIs allowed us to determine exactly which neurons were producing which frequencies of the original pattern. If the most common scenario was that a single neuron reproduces the entire pattern, the overlap between the populations of neurons producing each component frequency would be very high. This was however not the case, and we found that following training there was a decrease in the overlap, indicating that different neurons were storing different frequencies.

In the experiments presented, we observed that the addition of blockers of synaptic transmission prevented the occurrence of reverberations. While this supports the idea that the reverberations are generated by microcircuits of neurons or neuronal circuits which are modulated by glial interactions, further study of the specific identity and connectivity of the component cells will be necessary. However, these data do suggest a fundamental, inherent property of the microcircuitry of CNS neurons is to separate complex time-dependent information streams into component frequencies, much like a Fourier transform. In a model of brain function, transforming streams of time-dependent neuronal firing patterns into the frequency domain affords the brain the ability to turn time-dependent events into a single “object” of information. This is a process already observed in many biological systems such as during auditory and visual processing and would provide the same information processing benefits to the more complex cognitive functions of the cortex and hippocampus. These data directly demonstrate the presence of these mechanisms and some of their fundamental properties, and we believe this paradigm will allow the continued investigation of the precise microcircuitry involved in the processing of information in the brain.

## Synthesis

Reviewing Editor: Nathalie Ginovart, University of Geneva Decisions are customarily a result of the Reviewing Editor and the peer reviewers coming together and discussing their recommendations until a consensus is reached. When revisions are invited, a fact-based synthesis statement explaining their decision and outlining what is needed to prepare a revision will be listed below. The following reviewer(s) agreed to reveal their identity: NONE.

The manuscript has undergone significant improvements aimed at addressing the majority of criticisms raised by reviewers. However, two major comments from the previous review persist in the revised manuscript: the need for appropriate statistical analysis of the data (especially as the language used in the manuscript suggest the use of statistics, which is not the case), and the inclusion of an additional control group (which should be inexpensive and quick to do) to strengthen the findings. Upon consultation with the two reviewers, they both agreed on the critical importance of conducting appropriate statistical testing to support the main conclusions of the work, thereby strengthening the overall quality and impact of the research.

Please find the reviewer's comments below:

Reviewer 1:

The authors addressed all my concerns and I recommend publication.

Reviewer 2:

Overall the manuscript has been significantly improved and my belief in the value of this work remains. Although I would encourage the authors to ensure that figures are in the correct order or at least labelled for the peer review process, when figures are out of order it makes it more time consuming to figure out what you are trying to communicate.

Now that the manuscript has been significantly improved for readability I have only two outstanding comments, both related to previous comments:

Previous comment 1: Thank you for providing additional details, however none of those are statistical tests. If you are using the phrase 'significant difference' or 'statistical analysis' etc, people will assume you mean statistically significant. I can't understand why you would not be conducting the appropriate inferential statistical tests as is standard in this field on the results. You have a rich dataset, doing a deeper analysis even just on the graphs presented would take only a day or two and significantly strengthen your manuscript. For example (just one example) are the differences in # post stim stimulation frequencies ISIs different between different patterns. They look like they are but the effect of these differences needs to be calculated. I would expect this work to do this analysis on most graphs presented.

Previous comment: I suggested last time that you had limited controls and I stand by this statement. You should add in some simple control groups. I would settle for a media only control that is stimulated and that does not show the engram pattern after stimulation. I appreciate it is a dull control to have, but it would convince that you're not facing a system issue and it is very easy to add this control. I would say this is at least as important as your pre-stimulation control. Ideally quantify the average differences in Figure 2 in the pre-stimulation vs poststimulation conditions to show the effect is as robust as you claim.
