# Supplementary figures and images for: Detection of Memory Engrams in Mammalian Neuronal Circuits
Source: eNeuro. 2024 Aug 2;11(8):ENEURO.0450-23.2024. doi: 10.1523/ENEURO.0450-23.2024 (PMC11307552; doi:10.1523/ENEURO.0450-23.2024)

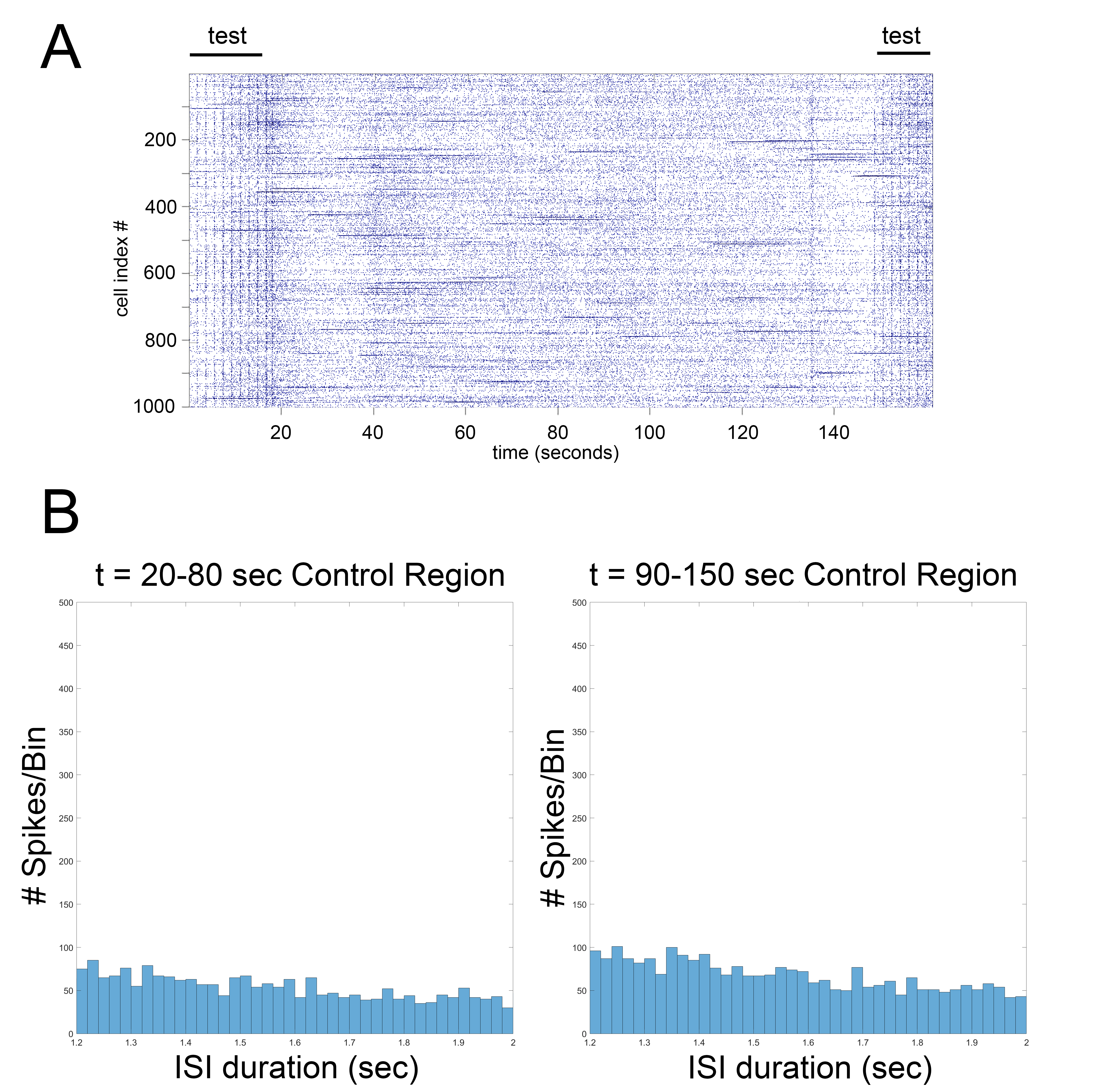

Supplement: Figure 3 — Language genetic influence result without a language mask A. Multiple types of whole-brain regions. After genetic modeling without restricting the language activation map, multiple types of regions were identified. B. The clustering result of the genetic regions without a language activation mask. Download Figure 3, TIF file. [file eneuro-11-ENEURO.0450-23.2024-s006.tif]
