# Supplementary material for: Detection of Memory Engrams in Mammalian Neuronal Circuits
Source: eNeuro. 2024 Aug 2;11(8):ENEURO.0450-23.2024. doi: 10.1523/ENEURO.0450-23.2024 (PMC11307552; doi:10.1523/ENEURO.0450-23.2024)
Supplement: Table 3A- 1a — Functional labels based on Neurosynth for genes contributing to commonality of language clusters Note: For each gene, the functional terms from Neurosynth represent the terms with the most similar meta-analysis whole-brain activation map to the gene’s whole-brain map. The correlation values indicate the correlation of the gene’s whole-brain expression with the term’s meta-analysis result. Download Table 3A, DOC file. [file eneuro-11-ENEURO.0450-23.2024-s002.doc]

**Table 3A – 1a**There was a statistically significant difference in the number of frequency-specific ISIs between the control and the 3 stimulation frequency patterns (3, 5, and 8 Hz (the first 4 columns of Figure 3A)), as determined by an ANOVA (F (3, 92) = 89.79, P<0.0001). To further investigate significant differences in the specific frequency patterns, Tukey post-hoc tests revealed that only the difference between the 3 Hz and the 5 Hz stimulation was not significant.

|  | Mean Difference | 95.00% diff. | P value |
| --- | --- | --- | --- |
| Control vs 3 Hz | -12.87 | -21.03 to -4.698 | P=0.0005 |
| Control vs 5 Hz | -24.44 | -31.79 to -17.08 | P<0.0001 |
| Control vs 8 Hz | -45.04 | -52.39 to -37.68 | P<0.0001 |
| 3Hz vs. 5Hz | -11.57 | -20.50 to -2.638 | P=0.0056 |
| 3Hz vs. 8Hz | -32.17 | -41.10 to -23.24 | P<0.0001 |
| 5Hz vs. 8Hz | -20.60 | -28.80 to -12.40 | P<0.0001 |
