# Supplementary material for: Detection of Memory Engrams in Mammalian Neuronal Circuits
Source: eNeuro. 2024 Aug 2;11(8):ENEURO.0450-23.2024. doi: 10.1523/ENEURO.0450-23.2024 (PMC11307552; doi:10.1523/ENEURO.0450-23.2024)
Supplement: Table 3A - 1b — Functional labels based on Neurosynth for genes contributing to differences of different language clusters Note: For each gene, the functional terms from Neurosynth represent the terms with the most similar meta-analysis whole-brain activation map to the gene’s whole-brain map. The correlation values indicate the correlation of the gene’s whole-brain expression with the term’s meta-analysis result. Download Table 3A - 1b, DOC file. [file eneuro-11-ENEURO.0450-23.2024-s003.doc]

**Table 3A – 1b**

ANOVA determined that the 8 Hz stimulation pattern and 8 Hz + CNQX, 8 Hz + APV, 8 Hz + KYNA, and 8 Hz + carbenoxolone/suramin (C/Sr) treatment (the last 5 columns of Figure 3A) were significantly different, (F (4, 51) = 9.615, P<0.0001). To further investigate the significance of the individual drugs, Tukey post-hoc tests revealed all treatments were significantly different.

|  | Mean Difference | 95.00% diff. | P value |
| --- | --- | --- | --- |
| 8 Hz vs 8 Hz + CNQX | 45.59 | 36.18 to 54.99 | P<0.0001 |
| 8 Hz vs 8 Hz + APV | 45.44 | 35.72 to 55.15 | P<0.0001 |
| 8 Hz vs 8 Hz + KNYA | 45.81 | 35.74 to 55.89 | P<0.0001 |
| 8 Hz vs 8 Hz + C/Sr | 42.86 | 29.02 to 56.71 | P<0.0001 |
