# Supplementary material for: Detection of Memory Engrams in Mammalian Neuronal Circuits
Source: eNeuro. 2024 Aug 2;11(8):ENEURO.0450-23.2024. doi: 10.1523/ENEURO.0450-23.2024 (PMC11307552; doi:10.1523/ENEURO.0450-23.2024)
Supplement: Table 3D - 1 — Download Table 3D - 1, DOC file. [file eneuro-11-ENEURO.0450-23.2024-s004.doc]

**Table 3D – 1

Statistical analysis of the ratio of excitatory cells to inhibitory cells.** Note: the primary goal of this data is to demonstrate that the ratio is biased toward the excitatory cells pre-stimulation and toward the inhibitory cells post-stimulation. T-test comparison resulted in all values being significantly different.

|  | t, df | F, DFn, Dfd | P value |
| --- | --- | --- | --- |
| E/I pre-stimulation | t=4.461, df=4 | 1.000, 2, 2 | P<0.0111 |
| E/I stimulated | t=3.134, df=4 | 1.000, 2, 2 | P<0.0351 |
| E/I post-stimulation | t=7.614, df=4 | 1.000, 2, 2 | P<0.0016 |
