# Supplementary material for: Detection of Memory Engrams in Mammalian Neuronal Circuits
Source: eNeuro. 2024 Aug 2;11(8):ENEURO.0450-23.2024. doi: 10.1523/ENEURO.0450-23.2024 (PMC11307552; doi:10.1523/ENEURO.0450-23.2024)
Supplement: Table 4J - 1 — Genetic model results for all the language genetic clusters after excluding specific object domain responsive voxels Note: For each language genetic cluster, the cognitive abilities with existing genetic effects (AE model or DE model, p values < 0.05, compared with the control model E, uncorrected) are marked with yellow. ΔAIC denotes the degree to which the best model is better than the control model. Download Table 4J - 1, DOC file. [file eneuro-11-ENEURO.0450-23.2024-s005.doc]

**Table 4J – 1**Statistical analysis of overlap between cell populations firing at component frequencies in multi-frequency stimulation. ANOVA comparing the overlap between neurons firing at 2 different frequencies during the control period, during the stimulation, and in the post-stimulation period showed a significant difference between the means (F(2,32) = 36.003, P < 0.0001). Tukey post-hoc analysis revealed that there was no significant difference between the control group and the stimulation group, but there was between all other comparisons.

|  | Mean Difference | 95.00% diff. | P value |
| --- | --- | --- | --- |
| control vs stimulation | 9.0 | -4.83 to 22.83 | P=0.2607 |
| control vs post stimulation | 29.7 | -42.97 to -16.43 | P<0.0001 |
| stimulation vs post stimulation | 45.81 | -50.44 to -26.96 | P<0.0001 |
